# Continuous vs. discontinuous purification of isolated human islets: functional and morphological comparison

**DOI:** 10.3389/fendo.2023.1195545

**Published:** 2023-06-30

**Authors:** Antoine Buemi, Nizar I. Mouard, Tom Darius, Arnaud Devresse, Nada Kanaan, Pierre Gianello, Michel Mourad

**Affiliations:** ^1^ Department of Surgery, Cliniques Universitaires Saint Luc, Université Catholique de Louvain, Surgery and Abdominal Transplantation Unit, Brussels, Belgium; ^2^ Pôle de chirurgie expérimentale et transplantation, Université catholique de Louvain, Brussels, Belgium; ^3^ Department of Internal Medicine, Nephrology Division, Cliniques Universitaires Saint-Luc, Brussels, Belgium

**Keywords:** islet isolation, islet purification, COBE 2991 cell processor, discontinuous density gradient, continuous density gradient

## Abstract

**Background:**

The COBE 2991 cell processor, commonly used for pancreatic islet isolation, is no longer distributed in Europe, leading to a search for alternative purification procedures with equivalent efficacy. The aim of this study was to evaluate the efficacy of an alternative method based on the discontinuous purification of islets.

**Methods:**

The conventional isolation procedure using a standard continuous islet purification with COBE 2991 of *n* = 4 human pancreas was compared to *n* = 8 procedures using a discontinuous purification with a “bottle” method from donors of similar characteristics. Islet equivalents, purity, and dynamic glucose-stimulated insulin secretion were evaluated.

**Results:**

A similar islet yield was obtained using continuous vs. discontinuous purification methods (76,292.5 ± 40,550.44 vs. 79,625 ± 41,484.46 islet equivalents, *p* = 0.89). Islets from both groups had similar purity (78.75% ± 19.73% vs. 55% ± 18.16%, *p* = 0.08) and functionality both in terms of stimulation index (3.31 ± 0.83 vs. 5.58 ± 3.38, *p* = 0.22) and insulin secretion (1.26 ± 0.83 vs. 1.53 ± 1.40 mean AUC, *p* = 0.73). Moreover, the size of the islets was significantly larger in the discontinuous vs. continuous purification group (19.2% ± 10.3% vs. 45.4% ± 18.8% of islets less than 100 µm, *p* = 0.0097 and 23.7% ± 5.3% vs. 15.6% ± 5.8% of 200–250 µm islet size, *p* = 0.03).

**Conclusion:**

Compared to the conventional purification procedure, discontinuous purification with a bottle method shows similar results with regard to isolation yield and islet secretory function. Furthermore, this alternative technique allows for obtaining larger islets.

## Introduction

Pancreatic islet transplantation is a minimally invasive treatment option to achieve better glucose control in selected patients with type 1 diabetes (1). The techniques used for islet isolation have improved over the years, leading to better outcomes. However, many recipients still require two- or three-islet transplantation procedures to achieve insulin independence (2). The purification process is a critical step in islet isolation, and its efficiency is directly related to the success of the procedure. Thus, up to 15%–51% rates of islet loss during this step have been reported (3).

Ficoll-based density gradients have been widely used for human islet purification in most islet processing centers using a semi-automated computerized COBE 2991 cell processor (4).

However, with the cessation of the distribution of COBE 2991 in Europe, there is an urgent need to investigate alternative purification methods.

Historically, other purification techniques have been used, such as handpicking, serial sieving, and discontinuous density gradient using 50–250 ml tubes/bottles, widely referred to as the “bottle” method (2, 4, 5). To our knowledge, the efficacy of these techniques has not been compared with the conventional COBE 2991 technique.

The present study aims to compare the efficacy of the COBE method vs. the bottle method for the purification of human islets from deceased human pancreas donors.

## Material and methods

Between November 2019 and May 2022, 36 human islet isolations were performed for research purposes.

Of these, *n* = 12 procedures were performed with the same functional and morphological assessment.

*N* = 4 human islet isolations were made using continuous purification, and *n* = 8 isolations were performed using discontinuous purification with the bottle method.

### Human pancreases for isolation

Human deceased donor pancreases were procured through a multiorgan donor program from 2019 to 2022 following aortic cross-clamp and *in situ* flush with Institut Georges Lopez-1 preservation solution (IGL-1) and transported to our islet laboratory facility. Organs were used for research only if discarded for clinical pancreas or islet transplantation and if research consent was present, according to the guidelines of our local medical ethical committee (Comité d’éthique hospitalo-facultaire UCL (CEHF), 2019/07MAI/201).

The following donor characteristics at donation were collected: donor age and gender, body mass index (BMI), graft origin, cause of death, medical history, laboratory results, medications, and ischemia times.

### Pancreatic islet isolation

Dissection, cannulation, enzymatic perfusion, and digestion of pancreatic tissue were identical in both groups. A blend of collagenase NB1 and neutral protease (SERVA Electrophoresis, Heidelberg, Germany) was used to perfuse the organ, aiming for pancreas distension with minimal leakage.

The enzyme was delivered *via* the pancreatic duct under controlled temperature and pressure. The perfused pancreas was then transferred to a Ricordi chamber and enzymatic and mechanical dissociation was initiated by warming the closed circuit to 37.0°C. Assessment of the tissue digest was performed under microscopy as timed samples were stained with dithizone, once a sufficient number of islets were free of exocrine tissue the digestion was halted by dilution of enzyme and washing of the tissue. The collected tissue was pooled and quantified to determine islet mass and the percentage of islets still encapsulated with acinar tissue.

#### Continuous purification

Islet separation from acinar tissue was achieved using a UW/Ficoll continuous gradient on a COBE 2991 cell processor. Density gradients of 1.062 and 1.072 g/ml were loaded into the COBE bag, followed by the addition of tissue digest suspended in UW solution. Following a 5-min centrifugation at 3,000 RPM, purity fractions were collected and again pooled to yield a pure layer and subsequent less pure layers.

#### Discontinuous purification

**Figure 1:**
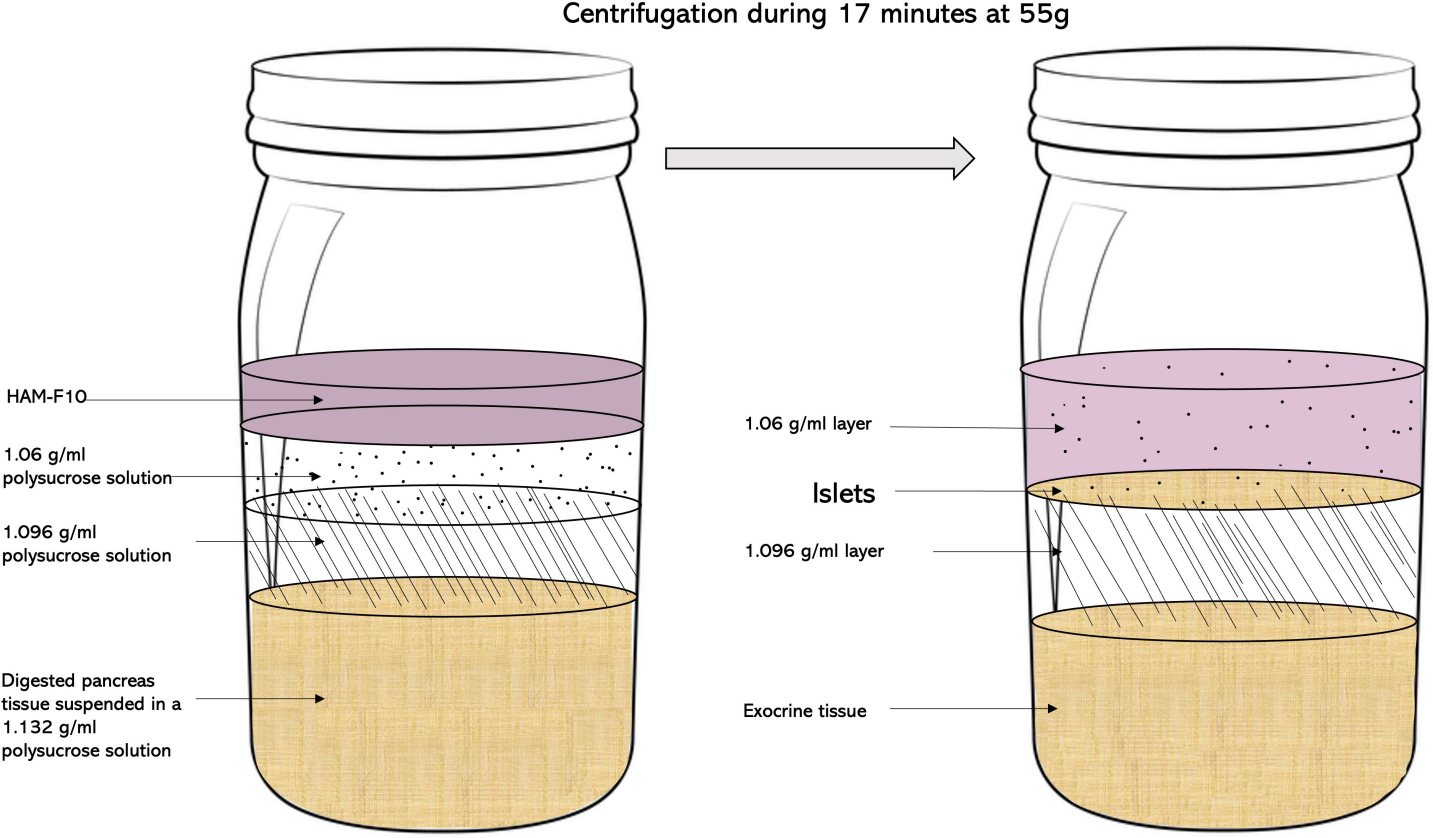
The “bottle” purification method. After the suspension of the digested pancreas tissue in a 1.132-g/ml polysucrose solution, two additional polysucrose solutions with densities of 1.096 and 1.06 g/ml, respectively, are added on top of the first layer to create a discontinuous density gradient. Isolated islets were then collected at the 1.096/1.06 interface following centrifugation for 17 min at 55 g.

Digested pancreas tissue was suspended in 75 ml of 1.132 g/ml polysucrose solution (Corning, USA) and transferred into a 250-ml flat-bottom bottle. Two additional polysucrose solutions with densities of 1.096 (65 ml) and 1.06 (45 ml) g/ml, respectively, were then carefully added by slowly pipetting the solutions on top of the first layer to create a discontinuous density gradient. All media were cooled to 4°C, and centrifugation was equally performed at this temperature. Isolated islets were then collected at the 1.096/1.06 interface following centrifugation for 17 min at 55 g (**Figure 1**).

### Islet assessment

#### Islet yield and purity

Quantification and purity of islets were performed at the end of the isolation using standard dithizone staining protocols (3). The crude number of islets in each diameter class was determined by counting islets using an optical graticule. The crude number of islets was then converted to the standard number of islet equivalents (IEQ).

#### Dynamic glucose-stimulated insulin secretion test

The secretory function of isolated human islets was assessed by dynamic islet perfusion experiments according to our previous protocols (6). The working medium was a bicarbonate-buffered solution containing 120 mM NaCl, 4.8 mM KCl, 2.5 mM CaCl_2_, 1.2 mM MgCl_2_, 24 mM NaHCO_3_, 1 mg/ml BSA, and varying concentrations of glucose as indicated in the figures. Batches of 1,000–2,000 IEQ were placed in perfusion chambers, covered with 8 µm cellulose filters, and sealed. Test solutions kept at 37°C and gassed continuously to stabilize pH around 7.2 were pumped at a flow rate of 1 ml/min. Effluent fractions were collected at 2-min intervals and saved for insulin assays using radioimmunoassay (RIA) kits (DiaSource ImmunoAssays, Ottignies, Belgium). At the end of the experiments, islets were recovered, and their insulin content was determined after extraction in acid-ethanol (75% ethanol, 180 mM HCl from Merck, Overijse, Belgium).

### Statistics

Data are presented as means ± standard deviation (SD). Differences between the two groups were analyzed using Student’s *t*-test. *p*-values < 0.05 were considered to indicate statistical significance.

## Results

### Donor characteristics

The donor characteristics of human pancreases that were used for islet isolation and the islet isolation outcomes are summarized in **Table 1**.

**Table 1:** Donor demographics, ischemia times and isolation data of 4 islet isolations using continuous purification compared with 8 islet isolations using discontinuous purification.

	Continuous Purification	Discontinuous Purification	P
	n=4	n=8	
Donor Demographics			
**Age, (years)**	52.2±20.6	48±10.7	0.64
**Gender, n**			0.71
**Male**	2	4	
**Female**	2	4	
**Weight (mean kg±SD)**	77±10.13	82.5±12.08	0.45
**Height (mean cm±SD)**	171±7.87	175.5±6,84	0.32
**BMI (mean kg/m^2^±SD)**	27±1.8	26,8±3.7	0.8
**Donor Organ Source, n**			0.99
DBD	1	2	
DCD	3	6	
**Cause of Death, n**			0.76
CVA	3	5	
Trauma	0	3	
Cardiac Arrest	1	0	
Cardiac Arrest, n	1	4	0.45
Medical History			
HBP, n	1	1	0.62
Smoking, n	0	3	0.99
Drug Abuse, n	0	1	0.99
Alchol Abuse, n	0	2	0.99
Infections, n	0	4	0.09
Vasopressor use, n	2	4	0.45
Laboratory Results			
Lipase, (mean IU/L)	31±24.57	40,.4±33.32	0.71
Amylase, (mean IU/L)	171±68.46	81.66±71.98	0.19
Hemoglobine, (mean g/dL)	13.37±1.37	11.01±2.20	0.07
Ischemia time			
Cold Ischemia time, h (±SD)	18.4±5.68	18.1 ±3.27	0.92
Warm Ischemia time, min (±SD)	12.1±20.2	20.37±9.07	0.48
Total Ischemia time, h (±SD)	19.23±5.53	18.1±3.29	0.72
Isolation data			
Islet yield (mean IEQ,±SD)	76292.5±40550.44	79625±41484.46	0.89
Islet purity(mean %,±SD)	78.75±19.73	55±18.16	0.08
IEQ/gr (mean IEQ/gr,±SD)	1181.6±650.5	753.5±397.7	0.18
100/150 size (mean %)	45.5±18.8	19.2±10.3	0.0097
150/200 size (mean %)	20.4±7.1	26.1±6.3	0.84
200/250 size (mean %)	15.6±5.8	23.7±5.3	0.0381
250/300 size (mean %)	9.6±3.2	11.8±5.3	0.47
300/350 size (mean %)	3.6±3.5	7.8±4.6	0.14
350/ size (mean %)	5.2±2.3	9.3±8.6	0.38

BMI, body mass index; CVA, Cerebrovascular Accident; DBD, donation after brain death; DCD, donation after circulatory death; IEQ, islet equivalent, HBP, high blood pressure; SD, standard deviation.

Baseline characteristics were similar in both groups.

### Isolation results

A similar islet yield was obtained using the continuous vs. discontinuous purification method (76,292.5 ± 40,550.44 vs. 79,625 ± 41,484.46, *p* = 0.89), even after correction for pancreas mass (1,181.68 ± 650.5 IEQ/g vs. 753.54 ± 397.73 IEQ/g, *p* = 0.18). The islet purity was also similar in continuous vs. discontinuous purification method groups (78.75% ± 19.73% vs. 55% ± 18.16%, *p* = 0.08).

Although the mean islet equivalent/g was similar in both groups (*p* = 0.18), some significant differences were found in the size of islets obtained after purification. Indeed, the percentage of islets with a size < 100 µm was higher in the continuous vs. discontinuous purification method groups (45.4% ± 18.8% vs. 19.2% ± 10.3%, *p* = 0.0097). In contrast, the percentage of islets measuring between 200 and 250 µm was lower in the continuous vs. discontinuous purification method groups (15.6% ± 5.8% vs. 23.7% ± 5.3% of islets size, *p* = 0.03). Case-by-case donor demographics results, ischemia times, and isolation data of both groups are available in the supplementary material (**Supplementary Tables S1**, **S2**).

**Figure 2:**
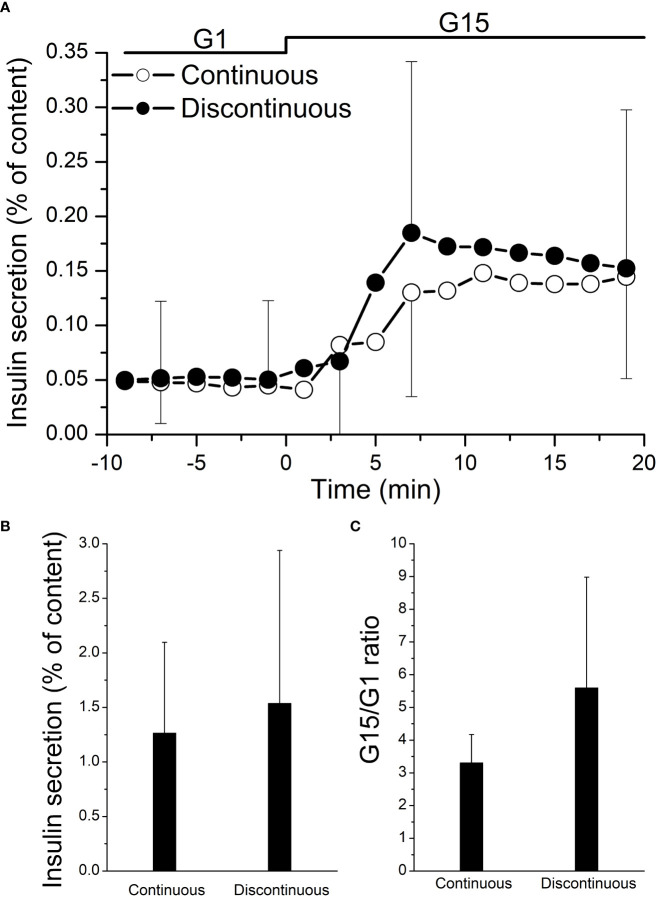
Functional testing of isolated human islets after pancreas purification with a continuous (*n* = 4) vs. a discontinuous (*n* = 8) method. On day 1, cultured islets from every isolation were perfused with a low concentration of glucose (1 mM glucose (G1)), followed by a high concentration of glucose (15 mM glucose (G15)). **(A)** Both the areas under the curve of insulin secretion (AUC) and stimulation index of each paired group were not statistically significant **(B, C)**.

After 1 day of culture, a dynamic GSIS was performed. Both islet groups were responsive to glucose stimulation, with a similar insulin secretory profile observed in islets purified by either method (**Figure 2A**): a sharp and rapid short-lived increase of insulin secretion (first phase) followed by a lower but constant secretion rate (second phase). The peak stimulation index was also similar in islets purified with a continuous vs. discontinuous purification method (3.31 ± 0.83 vs. 5.58 ± 3.38, *p* = 0.22) (**Figure 2B**). Also, the area under the insulin curve (calculated over 20 min of high glucose stimulation) was similar (1.26 ± 0.83 vs. 1.53 ± 1.40 mean AUC, *p* = 0.73) (**Figure 2C**).

## Discussion

Our results show that, compared to the conventional COBE 2991 isolation procedure, a technique using a discontinuous purification with a bottle method has similar performance in the isolation process and islet secretory function. If confirmed in larger-scale studies, these preliminary results suggest that this procedure might be considered an efficient alternative to the purification process of the human pancreas.

However, we acknowledge that even if similar, the results of the isolation process were poor in both groups, particularly in terms of the obtained islet yields.

As expressed in numerous international clinical studies, the most valuable parameters for deciding about the transplantability of isolated islets are the number of islets (generally they must correspond to more than 5,000 IEQ/kg of the recipient’s body weight), purity (as a percentage of insulin-positive clusters, generally > 70%), sterility, potency expressed by a stimulation index > 1, and viability of more than 70% (7). In our study, these values are not obtained due to the long ischemia times and the nature of the donors rejected for clinical purposes. Long ischemia times, both cold and warm, and donor unfavorable characteristics affect cell life/death, thereby influencing islet and acinar cells and, subsequently, isolation outcomes (8).

However, in both groups, the islet function remains satisfactory regardless of the type of purification method performed, both in terms of stimulation index and in terms of insulin secretion (9). We can conclude that the discontinuous purification method, by not impacting islet function, can replace continuous purification and that to obtain results compatible with a transplantable product, it is necessary to respect donor selection criteria and to minimize organ ischemia time.

Moreover, as previously described by others (10), the size of islets purified by the bottle method was significantly larger than that of islets purified by COBE purification. This is more likely related to the reduced shear stress during centrifugation in the purification using the bottle method. The COBE bag has a narrow segment, and islets suffered significant shearing force during passage through this narrow segment. Since the bottle does not have such a narrow segment, the shear stress could be significantly reduced.

The impact of islet size on islet transplantation outcomes is a debated topic in the literature.

Evidence has shown that at similar islet equivalent (IE) doses, larger islets may exhibit poorer therapeutic values. This may be related to oxygen diffusion limitations that worsen proportionally with islet size (11).

However, Hughes et al. have recently shown no clear correlation between islet isolation index and graft function in recipients receiving islets that had been cultured before implantation and that large islets are equally suitable as long as they have undergone and survived a short period of postculture isolation (12).

Moreover, in our experience, the bottle purification method was relatively simple to implement and associated with lower costs than the COBE 2991 method.

Our study has limitations. The first is the relatively low number of organs used. Second, because the protocol of the study was designed for research only, our results cannot be translated for clinical use.

In conclusion, we have demonstrated that discontinuous purification using the bottle method is a viable and efficient alternative in the absence of COBE 2991 that achieves comparable results both in terms of insulin secretory function and the performance of the isolation process from discarded organs.

The use of this purification technique with better-quality organs and shorter ischemia times would make it possible to permanently replace the islet purification gold standard of the past 20 years.

## Data availability statement

The raw data supporting the conclusions of this article will be made available by the authors, without undue reservation.

## Author contributions

AB and NM: study design and writing of the manuscript. They had equally participated in the elaboration of the paper. All authors discussed and reviewed the manuscript. All authors contributed to the article and approved the submitted version.
