# Supplementary material for: Continuous vs. discontinuous purification of isolated human islets: functional and morphological comparison
Source: Front Endocrinol (Lausanne). 2023 Jun 30;14:1195545. doi: 10.3389/fendo.2023.1195545 (PMC10348810; doi:10.3389/fendo.2023.1195545)
Supplement: Supplementary file 1 [file Table_1.docx]

|  | **Age (year)** | **Gender** | **Weight (Kg)** | **Height**  **(cm)** | **BMI** | **Category donor** | **Cause of death** | **Cardiac arrest** | **HBP** | **Diabetes** | **Smoking** | | **Drug Abuse** | | **Alcohol Abuse** | **Malignancy** | **Vasopressor Use** | **Infections** |
| --- | --- | --- | --- | --- | --- | --- | --- | --- | --- | --- | --- | --- | --- | --- | --- | --- | --- | --- |
| **Continous Purification Group** |  |  |  |  |  |  |  |  |  |  |  |  | |  | |  |  |  |
| **Case 1** | 27 | Male | 90 | 180 | 28 | DCD | CVA | Yes | No | No | No | No | | No | | No | Yes | No |
| **Case 2** | 44 | Female | 68 | 166 | 25 | DBD | CVA | No | No | No | No | No | | No | | No | No | No |
| **Case 3** | 67 | Female | 70 | 163 | 26 | DCD | Cardiac Arrest | No | No | No | No | No | | No | | No | No | No |
| **Case 4** | 71 | Male | 80 | 175 | 29 | DCD | CVA | No | Yes | No | No | No | | No | | No | No | No |
|  |  |  |  |  |  |  |  |  |  |  |  |  | |  | |  |  |  |
| **Discontinous Purification Group** |  |  |  |  |  |  |  |  |  |  |  |  | |  | |  |  |  |
| **Case 1** | 45 | Male | 93 | 180 | 29 | DCD | Trauma | No | No | No | Yes | Yes | | Yes | | No | No | No |
| **Case 2** | 43 | Female | 79 | 176 | 26 | DCD | CVA | No | Yes | No | Yes | No | | No | | No | No | No |
| **Case 3** | 30 | Male | 75 | 180 | 23 | DBD | CVA | Yes | No | No | No | No | | No | | No | Yes | Yes |
| **Case 4** | 53 | Male | 80 | 180 | 25 | DBD | CVA | Yes | No | No | No | No | | No | | No | Yes | No |
| **Case 5** | 45 | Female | 75 | 160 | 29 | DCD | CVA | No | No | No | Yes | No | | Yes | | No | Yes | Yes |
| **Case 6** | 60 | Male | 94 | 175 | 31 | DCD | Trauma | No | No | No | No | No | | No | | No | No | Yes |
| **Case 7** | 64 | Female | 64 | 173 | 21 | DCD | CVA | Yes | No | No | No | No | | No | | No | Yes | No |
| **Case 8** | 44 | Male | 100 | 180 | 31 | DCD | Trauma | Yes | No | No | No | No | | No | | No | No | Yes |

**Table S1**

**Donor demographics of 4 islet isolations using continuous purification compared with 8 islet isolations using discontinuous purification**

**Abbreviations:** BMI, body mass index; CVA, Cerebrovascular Accident; DBD, donation after brain death; DCD, donation after circulatory death; IEQ, islet equivalent, HBP, high blood pressure
