# Supplementary material for: Continuous vs. discontinuous purification of isolated human islets: functional and morphological comparison
Source: Front Endocrinol (Lausanne). 2023 Jun 30;14:1195545. doi: 10.3389/fendo.2023.1195545 (PMC10348810; doi:10.3389/fendo.2023.1195545)
Supplement: Supplementary file 2 [file Table_2.docx]

**Table S2**

**Ischemia times and isolation data of 4 islet isolations using continuous purification compared with 8 islet isolations using discontinuous purification**

|  | **Ischemia Times** | | | **Isolation Data** | | | | | | | | | | | |
| --- | --- | --- | --- | --- | --- | --- | --- | --- | --- | --- | --- | --- | --- | --- | --- |
|  | **CIT, h,min** | **WIT, min** | **TIT, h,min** | **IEQ** | **Purity (%)** | **Organ Weight (gr)** | | **IEQ/gr** | **100/150 (%)** | | **150/200 (%)** | **200/250 (%)** | **250/300 (%)** | **300/350 (%)** | **350 /(%)** |
| **Continous Purification Group** |  |  |  |  |  | |  |  | |  |  |  |  |  |  |
| **Case 1** | 23,46 | 16 | 24,02 | 34750 | 85 | | 100 | 409 | | 68,8 | 10 | 10 | 6,4 | 1 | 3,6 |
| **Case 2** | 23,06 | 0 | 23,06 | 54840 | 50 | | 78 | 703 | | 30,9 | 21,8 | 22,7 | 12,1 | 3,8 | 8,3 |
| **Case 3** | 12,52 | 16 | 13,08 | 127320 | 85 | | 77 | 1654 | | 29,4 | 26,2 | 18,18 | 12,8 | 8,5 | 5,8 |
| **Case 4** | 14,54 | 15 | 15,09 | 88260 | 95 | | 45 | 1961 | | 52,89 | 23,6 | 11,8 | 7,2 | 1,1 | 3,1 |
|  |  |  |  |  |  | |  |  | |  |  |  |  |  |  |
| **Discontinous**  **Purification Group** |  | |  |  |  | |  |  | |  |  |  |  |  |  |
| **Case 1** | 19,26 | 24 | 19,50 | 26760 | 45 | | 118 | 227 | | 24,5 | 37,4 | 25,4 | 7 | 2,2 | 3,2 |
| **Case 2** | 24,56 | 15 | 25,11 | 139960 | 55 | | 109 | 1284 | | 14,1 | 22,1 | 29,8 | 18,1 | 9,4 | 6,3 |
| **Case 3** | 18,08 | 5 | 18,13 | 66420 | 72 | | 85 | 781 | | 17,2 | 26,1 | 26,1 | 12,3 | 6,6 | 12,3 |
| **Case 4** | 16,38 | 25 | 17,03 | 125100 | 30 | | 149 | 840 | | 36,2 | 13,8 | 16,4 | 5,5 | 8,3 | 5,7 |
| **Case 5** | 17,24 | 21 | 17,45 | 83400 | 67 | | 94 | 887 | | 7,5 | 14,7 | 20,6 | 9,7 | 17,7 | 29,5 |
| **Case 6** | 14,31 | 31 | 15,02 | 55440 | 42 | | 131 | 423 | | 26,1 | 38,4 | 16 | 10 | 3,8 | 5,4 |
| **Case 7** | 17,50 | 30 | 18,20 | 105900 | 55 | | 85 | 1246 | | 4,8 | 33,9 | 29 | 21,2 | 7 | 3,9 |
| **Case 8** | 14,24 | 12 | 14,36 | 34020 | 72 | | 100 | 340 | | 23,4 | 22,7 | 26,5 | 11,3 | 7,5 | 8,3 |

**Abbreviations:** CIT, Cold ischemia time; IEQ, Islet equivalent; TIT, Total ischemia time; WIT, warm ischemia time;
